# Correction: Enhanced photoluminescence of silicon quantum dots in the presence of both energy transfer enhancement and emission enhancement mechanisms assisted by the double plasmon modes of gold nanorods

**DOI:** 10.1039/d1na90098f

**Published:** 2021-10-22

**Authors:** Jiahao Cao, Hanjie Zhang, Xiaodong Pi, Dongsheng Li, Deren Yang

**Affiliations:** State Key Laboratory of Silicon Materials, School of Materials Science and Engineering, Zhejiang University Hangzhou Zhejiang 310027 People’s Republic of China mselds@zju.edu.cn mseyang@zju.edu.cn

## Abstract

Correction for ‘Enhanced photoluminescence of silicon quantum dots in the presence of both energy transfer enhancement and emission enhancement mechanisms assisted by the double plasmon modes of gold nanorods’ by Jiahao Cao *et al.*, *Nanoscale Adv.*, 2021, **3**, 4810–4815, DOI: 10.1039/D1NA00287B.

This article was originally published without ESI. This correction notice is to notify readers that ESI has now been uploaded with the article.

The Royal Society of Chemistry apologises for any inconvenience to authors and readers.

## Supplementary Material

